# The role of Lactobacillus in inflammatory bowel disease: from actualities to prospects

**DOI:** 10.1038/s41420-023-01666-w

**Published:** 2023-09-29

**Authors:** Congxin Li, Kaixin Peng, Siqi Xiao, Yuanyuan Long, Qin Yu

**Affiliations:** 1 Department of Gastroenterology, Tongji Hospital of Tongji Medical College, Huazhong University of Science and Technology, Wuhan, P. R. China; 2 Institute of Liver and Gastrointestinal Diseases, Tongji Hospital of Tongji Medical College, Huazhong University of Science and Technology, Wuhan, P. R. China

**Keywords:** Inflammatory bowel disease, Microbiology

## Abstract

Inflammatory Bowel Disease (IBD), a chronic nonspecific intestinal inflammatory disease, is comprised of Ulcerative Colitis (UC) and Crohn’s Disease (CD). IBD is closely related to a systemic inflammatory reaction and affects the progression of many intestinal and extraintestinal diseases. As one of the representative bacteria for probiotic-assisted therapy in IBD, multiple strains of Lactobacillus have been proven to alleviate intestinal damage and strengthen the intestinal immunological barrier, epithelial cell barrier, and mucus barrier. Lactobacillus also spares no effort in the alleviation of IBD-related diseases such as Colitis-associated Colorectal cancer (CAC), Alzheimer’s Disease (AD), Depression, Anxiety, Autoimmune Hepatitis (AIH), and so on via gut-brain axis and gut-liver axis. This article aims to discuss the role of Lactobacillus in IBD and IBD-related diseases, including its underlying mechanisms and related curative strategies from the present to the future.

## Facts

- Lactobacillus is a representative of probiotic-assisted therapy in IBD and is approved to alleviate colitis in both mice with DSS-induced colitis and IBD patients.
- IBD was pointed out to impact the systemic inflammatory reaction, leading to the aggravation of other extraintestinal diseases, especially in the liver and CNS.
- The combination of Lactobacillus and other probiotics or prebiotics is widely used in treating IBD in clinical trial stages, and some of them have apparent therapeutic effects.

## Open questions

- What are the specific mechanisms Lactobacillus possesses to alleviate IBD?
- What role does Lactobacillus play in IBD-related diseases in the gut-brain and gut-liver axes?
- Does the effect of probiotics change with the degree of inflammation in IBD and IBD-related diseases?
- How can we reasonably combine the use of probiotics and prebiotics in treating IBD and IBD-related conditions to maximize the effectiveness of Lactobacillus?

## Introduction

Inflammatory Bowel Disease (IBD), a chronic nonspecific intestinal inflammatory disease that generally causes abdominal pain, diarrhea, and bloody stools, displayed its globalization from Western developed regions to newly industrialized countries in the twenty-first century [1]. IBD is comprised of ulcerative colitis (UC) and Crohn’s Disease (CD), the former mainly causing successional shallow ulcers with intestinal crypt abscesses in lamina propria and the latter represented by segmental distributed deep ulcers accompanied with non-caseating granulomas in all layers of the intestinal wall which explained its high tendentiousness in fistulation [2]. The existing treatment of IBD focused on amino-salicylic acid preparation, glucocorticoids, immunosuppressants, and new-emerging biological agents such as infliximab and adalimumab. Though possessing low mortality, frequent relapse, and high disability rate of IBD made it a heavy burden to not only public health but also the fiscal and resources in healthcare systems, announcing the urgent demand to explore applicable therapeutic methods to alleviate the misery of IBD patients in both active and remission period.

Gut microbiota, consisting of bacteria, archaea, fungi, viruses, and parasites, was closely related to intestinal disease occurrence, development, and prognosis [3]. Among the above microbes, research in the field of human intestinal bacteria, mainly including Firmicutes, Bacteroidetes, Actinobacteria, Fusobacteria, Proteobacteria, Verrucomicrobia, and Cyanobacteria, were booming and showed terrific development prospects in recent years [4]. Within the considerable collective of intestinal bacteria, Lactobacillus, a prominent member of the Firmicutes, caused widespread concern as its subpopulations were put forward to affect the deterioration of course in IBD patients as well as to display its protective effect in the experimental colitis mouse model [5]. Despite the significant talent lactobacillus unveiled, summaries to wrap up its particular underlying mechanism of action in IBD and IBD-related diseases were limited. Therefore, we are here to promulgate the role of Lactobacillus in IBD and IBD-related disorders and the microbiota-gut-brain axis (MGBA) to propose our insights.

## The subspecies of Lactobacillus and its relationship with IBD

Lactobacillus, a genus of gram-positive anaerobic bacteria without spores, was named after its capability to decompose glucose and other sugars into lactic acid. As a member of Firmicutes, it belonged to the class Bacillus, order Lactobacillales, family Lactobacillus. It was comprised of various subspecies, including Lactobacillus acidophilus, Lactobacillus salivarius, Lactobacillus plantarum, Lactobacillus casei, Lactobacillus rhamnosus, Lactobacillus gasseri, and so on [6]. According to available data, it was estimated that this genus accounted for about 0.3% of the total bacterial count in the human colon, while when it comes to human duodenum, the figure reached up to 6% [7, 8]. Based on experimental research in Lactobacillus and mice with experimental colitis so far, the majority of Lactobacillus’ subspecies did good to the intestinal health and helped with the restoration of the host from excessive intestinal inflammation as what we would put later, while only a fraction played the opposite conducts. As for the latter, for example, it has been observed that the supernatant of Lactobacillus delbrueckii CU/22 could lead to the apoptosis and necrosis of HT-29 cells, suggesting that careful consideration ought to be involved in choosing probiotics as adjuvant therapy of IBD [9].

When we looked at the clinical analysis, there was a positive correlation between intestinal Lactobacillus abundance and clinical symptoms in UC patients, as expected, while the situations in CD patients were still under dispute [5, 10, 11]. Consequently, great importance ought to be attached to proclaiming the role of Lactobacillus in IBD.

## The mechanism underlying the barrier-protective effect of Lactobacillus in IBD

### The effect of Lactobacillus on the immunological barrier

#### The effect of Lactobacillus on immune cells

As the first line of defense against microbiota, intestinal mucosa harbors numerous immune cells consisting of lymphocytes, dendritic cells, granulocytes, etc. Their methodical interactions with each other maintain immune homeostasis in both peripheral blood and intestinal mucosal microenvironment. However, under colitis, such tacit understanding cooperation received severe interference. In patients with IBD, there was proved to exist a pathological transformation from Tregs to Th17 in peripheral blood, and the subsequently reduced Treg/Th17 ratio observed in both adults and pediatric patients may, to a certain extent, explain the hampered systemic immunosuppressive function [12–14]. Besides, though possessing specific divergence in the subtype of numerous immune cells, UC and CD patients were recorded to display higher neutrophil-to-lymphocyte ratio (NLR) in peripheral blood compared to healthy controls [15]. Regarding the immune microenvironment in the inflamed colonic tissue, the frequency of M1-like macrophages, activated DCs, plasmacytoid DCs, and monocytes increased as well [16]. To summarize, the hyperfunction of pro-inflammatory cells and the low vitality of anti-inflammatory cells resulted in disturbed immune homeostasis, which could be a prospective reversible therapeutic target for IBD.

The aptitude of Lactobacillus to restore the altered immune cells proportion was proved in many pieces of research, which pointed out the strengthened anti-inflammatory force and hindered pro-inflammatory power after the administration of Lactobacillus strains such as Lactobacillus reuteri and Lactobacillus rhamnosus. Respectively, Lactobacillus reuteri was able to not only prevent the recruitment of neutrophils and the expansion of DCs in the intestinal mucosa but also increase the frequency of Tregs in mesenteric lymph nodes which were the supreme headquarters of intestinal immune cells [17]. Apart from Lactobacillus reuteri, Lactobacillus rhamnosus was also competent in interfering with the relative abundance of immune cells since it was proved to decrease the Th17/Treg ratio through the JAK-STAT signaling pathway with the presence of toll-like receptor 2 (TLR2) in the colon of mice with DSS-induced colitis [18]. In addition, it has been uncovered that soluble factors that existed in the supernatants of Lactobacillus rhamnosus could promote the mitochondrial pathway-dependent apoptosis of specific immune cells, mainly monocytes, without affecting intestinal epithelial cells (IECs), indicating its desirable value in the prevention of excessive inflammatory activation [19].

Except for changing the number and proportion of different immune cells, the phenotype of immune cells was also transformed by Lactobacillus. As a vital constituent of antigen-presenting cells (APCs), macrophage possesses two exact opposite-in-function phenotypes, M1 and M2, the former polarized by lipopolysaccharide (LPS), producing pro-inflammatory cytokines such as IL-1β, IL-6, IL-12, IL-23, and TNF-α, the latter polarized by Th2 cytokines, producing anti-inflammatory cytokines such as IL-10 and TGF-β [20]. Lactobacillus reuteri GroEL, a kind of hot shock protein (HSP) homologous with human HSP60, was found to regulate the phenotypic modulation of macrophages concretely embodied in that it inhibited M1-like macrophage markers while enhancing the expression of M2-like macrophage markers by how it relieved the inflammatory pressure in mice [21]. Except for Lactobacillus reuteri, Lactobacillus rhamnosus Lr32 and Lactobacillus salivarius Ls33 protected mice from TNBS-induced colitis by promoting DCs’ differentiation to a specific tolerogenic phenotype featured by their inability neither to produce cytokines or chemokines nor to express co-stimulatory molecules to activate T cells [22]. More precisely, these peculiar DCs conveyed their anti-inflammatory capability by reducing the expression of pro-inflammatory mediators containing IL-17 and IL-23 and negatively regulating T-cell function via overexpressing indoleamine 2, 3 dioxygenases (IDO) [22].

In the above research outcomes, Lactobacillus interfered with the excessive activation of intestinal and body inflammatory reactions in multiple directions and angles by changing the number, recruitment, and differentiation of immune cells, thus limiting the development and exacerbation of IBD.

#### The effect of Lactobacillus on inflammatory cytokines and underlying mechanisms

Dialectically, there was a correlation between immune cell distribution and the secretion of inflammatory cytokines since numerous immune cells secreted inflammatory cytokines, which could, in turn, interfere with their recruitment and function. Just as we derived it from the previous section, various Lactobacillus strains represented by Lactobacillus rhamnosus [23], Lactobacillus jensenii [24], Lactobacillus reuteri [17], Lactobacillus casei [25], and Lactobacillus plantrum [26], downregulated the production of pro-inflammatory mediators such as interleukin-6 (IL-6), interleukin-1β (IL-1β), tumor necrosis factor-α (TNF-α) and otherwise in the inflamed tissues of colitis mice. Meanwhile, the expression of anti-inflammatory mediators was upregulated by Lactobacillus as well. A pleiotropic factor played a role in immunomodulation, G-CSF, negatively associated with pro-inflammatory mediators such as TNF-α, IL-23, and IL-12, was constitutively expressed at a high level in intestinal lamina propria cells both in mouse and healthy individuals, but at a relatively low level in IBD patients [27]. Lactobacillus rhamnosus GR-1, according to Andrew J Martins and his colleagues, could escalate the expression of G-CSF in healthy individuals but failed to reverse G-CSF reduction in IBD patients, thus exhibited their prophylaxis but not treatment value [27].

The mechanisms were diligently explored after observing that the lactobacillus mentioned above hampered the production and secretion of inflammatory mediators. The pattern recognition receptor (PRR) family was elucidated instrumental in the anti-inflammatory effect of various strains. To illustrate, by contacting with toll-like receptor 4 (TLR4) and inhibiting its downstream TLR4/Myd88/NF-κB signaling axis in colon tissues of mice, a novel soluble protein HM0539 from Lactobacillus rhamnosus downregulated the expression of cyclooxygenase-2 (COX-2) and inducible nitric oxide synthase (iNOS), therefore hindered the production of prostaglandin E2 (PGE2) and nitric oxide (NO), two vital inflammatory mediators in the gut [23]. Let aside the TLR family, the nucleotide-binding oligomerization domain (NOD) family also mediated robust recognition. NOD2 was elucidated to identify strain-specific muropeptides secreted from peptidoglycan (PGN), a cell-wall component of Lactobacillus, to activate different response programs of immune cells and lead to quite the opposite influence in the production of an assortment of cytokines [28].

While hindering the production of inflammatory cytokines, Lactobacillus could also accelerate the degradation of tissue-distributed inflammatory-related factors. Interferon-inducible protein-10 (IP-10), a proinflammatory chemokine whose combination with its receptor CXCR3 activated multiple signaling pathways to recruit lymphocytes and lead to the influx of inflammatory cytokines into inflamed mucosa, could be selectively degraded by lactocepin, a protease encoded by Lactobacillus casei or Lactobacillus paracasei. Meanwhile, Lactobacillus casei-fed mice with colitis revealed a significantly interfered infiltration of T cells and ameliorated inflammation in cecal tissue compared to the control group [29]. Coincidentally, IP-10 was also noticed in another research where the degradation was explained as that Lactobacillus casei could hinder the vesicle transport of IP-10 to the cell membrane, which was crucial as a prerequisite for the function of IP-10 [30].

In general, Lactobacillus changed the downstream signal pathway by combining with specific recognition receptors, thus affecting the release and degradation of inflammatory factors to alleviate intestinal inflammation ultimately.

### The effect of Lactobacillus on intestinal epithelial cell barrier

Tight junctions (TJs) widely exist in the lateral surface of IECs near the luminal side of the intestine, conducted as important complexes to seal the spaces between IECs, maintain cell polarity, and sustain the permeability barrier. TJs comprised transmembrane proteins such as occludins and claudins and peripheral membrane proteins such as ZO (zonula occludens proteins) [31]. TJs were observed downregulated in mice with DSS-induced colitis, resulting in elevated intestinal mucosa permeability, which may cause bacteria translocation, ion exchange disorder, etc. However, the administration of a great variety of Lactobacillus salvaged this disaster. Lactobacillus acidophilus KLDS 1.0901, Lactobacillus plantarum KLDS 1.0318, and Lactobacillus helveticus KLDS 1.8701, as well as their mixture possessed excellent ability to restore the expression of TJs, including E-cadherin, ZO-1, occludin and claudin-1in mice with colitis [32]. In addition, as a probiotic mixture comprised of Bifidobacterium, Lactobacillus acidophilus, and Enterococcus, Bifico, which was demonstrated to induce the alleviation of both TNBS-induced experimental colitis in mice and IBD patients, also increased TJs expression in DSS-induced colitis in mice [33].

Heat shock protein (HSP), widely existing in all kinds of organisms ranging from bacteria to mammalities and showing high evolutionary conservation, guarantees the correct fold of proteins and protects cells from injuries such as hyperthermia and oxidative stress. In DSS-induced colitis, the expression of HSP is delineated to decrease, indicating the undermined stress protective mechanism in IECs. However, these unpleasant damages could be reversed by the peroral treatment with Lactobacillus reuteri as announced by the research that strain ATCC PTA 4659 displayed the capability to restore the expression of HSP25 and Hsp70 and subsequently help to maintain the integrity of the cytoskeleton and free IECs from oxidative injury in rodent model with colitis. In addition, ATCC PTA 4659 could also strengthen the barrier-preserving TJs between IECs, contributing to its anti-inflammatory effect [17]. As a supplement, soluble factors obtained from Lactobacillus GG were also revealed to participate in the upregulation of Hsp25 and Hsp72 in IECs with the indispensable help of P38 and JNK, two subtribes in the MAPK pathway [34].

Apart from HSP, antioxidant enzymes (AOEs), which were demonstrated to decrease in quantity in the case of enteritis, also played a role in cell protection against attack from oxygen radicals. To cite an example, superoxide dismutase (SOD), especially Mn-SOD, a representative of protective enzymes that neutralized reactive oxygen species (ROS), which were inducers of inflammation in various diseases, was measured reduced in inflamed tissue from IBD patients [35]. In addition to SOD, there exited glutathione peroxidase (GSH-Px), catalase (CAT), and so on in the serum and colonic tissue, devoted to insulating intestinal mucosa from oxidative injury. In this aspect, Lactobacillus plantarum ZS62, the oral administration of which alleviated the severity of DSS-induced colitis in mice, was proved to upregulate the levels of CAT and T-SOD in the serum, and the expression of Cu/Zn SOD, Mn SOD, GSH-Px, CAT in inflamed colon tissues [26]. As a supplement, Lactobacillus gasseri producing Mn-SOD ameliorated the inflammation in IL-10-deficient mice with distinctly decreased infiltration of neutrophils and macrophages into colitis tissue, too [36]. A similar effect emerged in Lactobacillus delbrueckii subsp. Bulgaricus B3 and Lactobacillus delbrueckii subsp. Bulgaricus A13 as well [37].

Under the circumstances of a wrecked epithelial barrier, IECs got injured. At the same time, extracellular matrix glycosaminoglycans (GAGs) were exposed to the intestinal microflora, released GAG-degrading enzymes comprised of chondroitinase, hyaluronidase, and tryptophanase, the productions of which could conversely exert a cytotoxic effect on IECs [38, 39]. At this point, such a vicious circle was broken by Lactobacillus plantarum HY115 and Lactobacillus brevis HY7401; both were detected to reduce the production of GAG-degrading enzymes mentioned above in gut microflora by how they protected IECs against injury, which may also explain the colitis alleviation to a certain extent [40].

In a word, various Lactobacillus strains spared no effort in protecting IECs via strengthening the intestinal tract barrier, upregulating HSP expression, enhancing the storage of AOEs, and reducing IEC-harmful products from intestinal microbiota.

### The effect of Lactobacillus on the intestinal mucus barrier

The intestinal mucus barrier, mainly comprised of mucins, covers the intestine’s surface, protecting IECs from toxic substances, digestive enzymes, and bacteria. Mucins were commonly divided into transmembrane mucin represented by Muc 3/4/12/13/17 and mucin able to form a gel, such as Muc 2, whose absence led to a higher trend of colitis and colon cancer [41, 42]. In LPS-treated mice, the expression of Muc-2 and Muc-4 was downregulated, which was reversed by supplement of Lactobacillus rhamnosus LAB3 and Lactobacillus plantarum LAB39, indicating the effect of the above two strains in protecting mucus barrier in LPS-induced colitis [43]. A new synbiotic, FCT, made of Lactobacillus gasseri 505 and cudrania tricuspidata leaf extract, successfully increased the expression of Muc-2 and TFF3 in mice. The same results were also obtained from Lactobacillus casei strain Shirota (LcS) and the mixture of Lactobacillus acidophilus KLDS 1.0901, Lactobacillus helveticus KLDS 1.8701, and Lactobacillus plantarum KLDS 1.0318 [32, 44]. Interestingly, by comparing the adhesion ability to mucin of microbiota ranging from lactobacillus over fecal coliforms, bifidobacteria, and clostridia to total anaerobes, an in vitro adhesion experiment pointed out that it was Lactobacillus rhamnosus GG that adhered most selectively to mucin, indicating its crucial role in maintaining the mucus barrier [45].

**Fig. 1:**
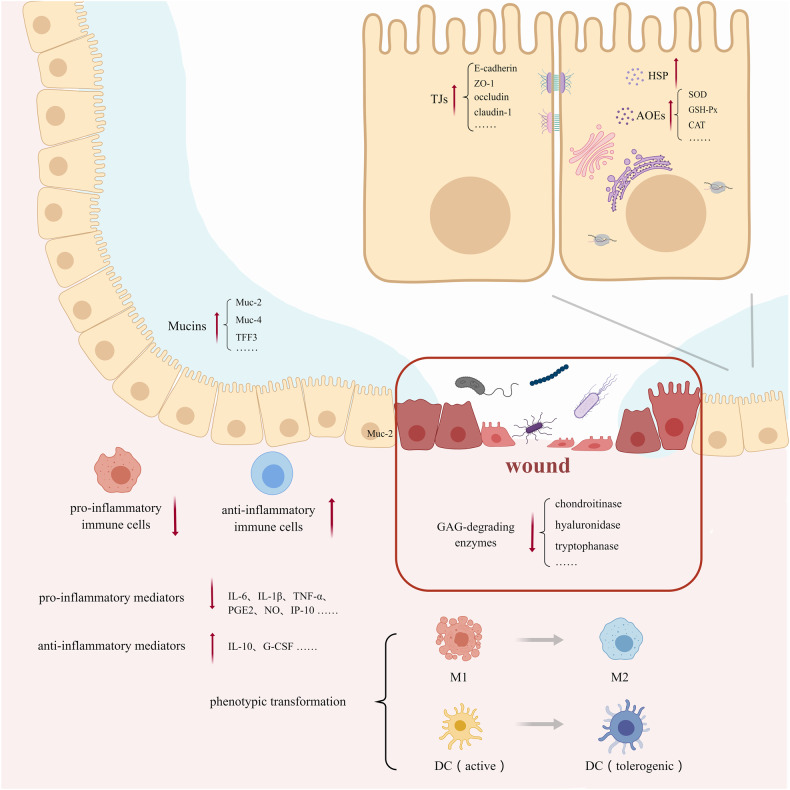
The mechanism underlying the barrier-protective effect of Lactobacillus in IBD. Lactobacillus maintained the intestinal immune barrier by altering the number of immune cells and the expression level of inflammatory factors. At the same time, it strengthened the cellular barrier by affecting the tight junction of intestinal epithelial cells and the expression level of intracellular protective proteins. Finally, it strengthened the mucus barrier by increasing the expression of protective mucin.

To better demonstrate our statement about the mechanism underlying the barrier-protective effect of Lactobacillus in IBD, a figure was shown here to summarize our point of view. (Fig. 1)

## The role of Lactobacillus in intestinal microbiota microenvironment in IBD

While the gut immune microenvironment of IBD patients was disrupted, their gut microbiota balance was also in a state of disorder. In detail, the abundance of Firmicutes, Verrucomicrobia, Akkermansia, and Lactobacillus, considered beneficial bacteria, decreased, while Bacteroidetes increased [46]. Due to the complexity of gut microbiota, the interaction between microorganisms in IBD should be taken seriously. Although the direct interaction between gut bacteria has not yet been fully elucidated, many microorganism supplements have been detected to affect the abundance of other intestinal flora in IBD-related studies. Therefore, it is inferred that they may have a synergistic and antagonistic relationship.

For example, the Lactobacillus plantarum Q7 supplement reduced the abundance of Proteobacteria, which was generally seen as pro-inflammatory bacteria, and increased the number of Bifidobacteria and Muribaculaceae, seen as anti-inflammatory genera [47]. The synergist of Lactobacillus and Bifidobacteria was also greatly important in another two studies focused on colitis and colitis-related depression [48, 49]. Aside from Lactobacillus and Bifidobacteria, different Lactobacillus strains, such as L. rhamnosus BY-02 and L. plantarum BY-05, also worked together and produced better results [50]. Moreover, it has been observed that Lactobacillus paracasei L9 improves colitis by expanding butyrate-producing bacteria such as Lachnospiraceae and Ruminococcaceae, which was observed reduced in colitis, indicating that they may have cooperation in treating colitis [11, 51].

Furthermore, the additional treatment of other bacteria may also influence the abundance of Lactobacillus. Pediococcus pentosaceus CECT 8330, a protective bacterium that could alleviate DSS-induced colitis, was found to elevate the quantity of Lactobacillus, Bifidobacterium as well as Dubosiella in mice intestine, suggesting that these bacteria may be in the same camp in alleviating enteritis [52]. Except for Pediococcus pentosaceus and Bacillus subtilis, Bifidobacterium bifidum was also proven to increase the amount of Lactobacillus and thus prevent colitis from exacerbating [53, 54].

When we mentioned the gut microenvironment of IBD patients, it was not only bacteria that should be considered. As we can anticipate, the intestinal condition of IBD patients is more complex than that of experimental colitis mice because of different microbial compositions and opportunistic infections caused by specific fungi. To better imitate the microenvironment in colitis, additional Candida albicans were colonized to the mouse gastrointestinal tract together with DSS administration as they do in the intestines of patients with colitis [55]. The results showed that Lactobacillus rhamnosus L34 could ameliorate gut local inflammation, gut-leakage severity, fecal dysbiosis, and systemic inflammation caused by opportunistic infection represented by Candida albicans in colitis, indicating the confrontation of Lactobacillus with pathogenic fungi to alleviate colitis in IBD [55].

## Chinese herbal medicine and food that could enhance colitis and alter the abundance of Lactobacillus in IBD

As a regular probiotic in the intestine, the abundance of Lactobacillus is easily affected by food and oral medication. As a chronic disease prone to recurrence and persistence, the importance of daily diet and traditional Chinese medicine treatment should not be ignored in treating IBD. In this field, many traditional Chinese medicines have been pointed out in laboratory research to improve the abundance of Lactobacillus in the intestine while alleviating enteritis, such as Ampelopsis, Lithospermum, Rhubarb and so on [56–58]. Meanwhile, food containing fructooligosaccharides, glycerol monolaurate, Glycine, etc., was also beneficial for IBD alleviation [59–61]. After showcasing the many benefits of Lactobacillus in enteritis, we summarized the Chinese herbal medicines, foods, and their active ingredients that can change the abundance of Lactobacillus in IBD-related research (Table 1).

**Table 1:** Chinese herbal medicines, foods, and their active ingredients that can change the abundance of Lactobacillus in IBD.

Chinese herbal medicine/Food	Ingredients	Effect on Lactobacillus	Effect on colitis
Ampelopsis	Dihydromyricetin	increase	alleviate
Bananas, garlic, wheat, onions, honey, etc	Fructooligosaccharides	increase	alleviate
Barley leaf	-	increase	alleviate
Blueberries, onions, grapes, etc	Quercetin	increase	alleviate
Breast milk and coconut oil	Glycerol monolaurate	increase	alleviate
Caesalpinia genus plants	Cassane diterpenoids	increase	alleviate
Dendrobium officinale	Polysaccharide	increase	alleviate
Human breast milk	Glycerol monolaurate	increase	alleviate
Lithospermum	Shikonin	increase	alleviate
Mango	Polyphenols	increase	alleviate
Meat, soy products, nuts, dairy products	Glycine	increase	alleviate
Millet	Selenylated soluble dietary fiber	increase	alleviate
Myrciaria jaboticaba peel	-	increase	alleviate
Purple sweet potato	Anthocyanin extract	increase	alleviate
Radish	Sulforaphene	increase	alleviate
Rhodiola rosea L.	Salidroside	increase	alleviate
Rhubarb	Rhein	increase	alleviate
Soy hulls	Soluble dietary fiber	increase	alleviate
Tea	Tea polysaccharides and tea polyphenols	increase	alleviate
Tea flowers	Polysaccharides	increase	alleviate
Vaccinium macrocarpon	-	increase	alleviate

## The role of Lactobacillus in IBD-related gut-liver axis

As the connections between different organs receive increasing attention, the gut-liver axis emerged as a new perspective for recognizing liver diseases. IBD and intestinal inflammation were addressed to be tightly associated with viral hepatitis, autoimmune hepatitis (AIH), primary biliary cirrhosis (PBC), and primary sclerosing cholangitis (PSC), with the link between regarded as gut commensals, pathogens, and intestinal antigens [62]. To illustrate, statistical data displayed that more than 75% of PSC patients simultaneously suffered from IBD, mostly UC [63]. A retrospective study on Chinese IBD patients revealed a higher prevalence of HBV infection in IBD patients than in non-IBD patients [64]. In addition, the application of immunosuppressive medicines in IBD caused a significantly higher trend of liver dysfunction in hepatitis virus carriers, especially the combined use of multiple immunosuppressants in HBV carriers [65]. Regarding HBV vaccination, the response rate to the HBV vaccination was also lower among IBD patients under anti-TNF therapy compared to healthy controls [66]. In terms of hepatocellular carcinoma, the upregulated inflammatory mediators also led to a positive correlation with the incidence rate of hepatocellular carcinoma in patients with IBD [67]. All the above revealed the close connection between IBD and liver diseases.

As a sterile organ, the liver is often the primary victim of intestinal inflammation accompanied by bacterial translocation. Therefore, in studies related to liver diseases, direct contact with bacteria often exacerbated the development of the disease, and Lactobacillus is no exception. Under intestinal inflammation, increased intestinal permeability allowed for bacterial translocation and passage from the portal vein into the liver, contributing to the pathogenesis of numerous diseases such as AIH and cholestatic liver disease. Specifically, the production of IL-17 by intrahepatic γδ T cells, pivotal in the exacerbation of cholestatic liver diseases, was observed up-regulated when γδ TCR+ cells were exposed to heat-killed Lactobacillus gasseri [68]. The same results were observed when Lactobacillus gasseri was intraperitoneally injected into mice, leading to increased serum levels of IL17 and inflammatory cell infiltration into the liver [68]. In AIH, it has been proved that a signaling pathway mediated by hydrocarbon receptor (AhR) was necessary for ongoing AIH-like pathology, and Lactobacillus reuteri could release an AhR ligand, indole-3-aldehyde (I3A), thus promoting the differentiation of CD8 T cells in vitro and AIH-like pathology in mice [69]. Therefore, hepatic translocation of Lactobacillus acted unfriendly to the outcome of liver diseases.

However, when the barrier function of the intestine was in a normal state or not wholly paralyzed, which indicated that the occurrence of bacterial translocation is not allowed, gratifying results emerged as the supplement of Lactobacillus in the gut could alleviate AIH severity through microbiota-gut-liver axis. To demonstrate, in experimental AIH, Lactobacillus rhamnosus GG supernatant treatment successfully limited liver damage with the existence of polymeric immunoglobulin receptors [70]. Meanwhile, in a clinical trial, the Lactobacillus supplement enhanced the suppressive effects of prednisone on the levels of clinical indexes in AIH patients [71]. After experiments in mice, the mechanism may be due to prednisone’s enhanced suppressive capability on Tfh cell proportion in peripheral blood monocytes through the TLR4/MyD88/NF-κB pathway after Lactobacillus application [71].

In summary, the impact of Lactobacillus on liver diseases under enteritis seemed to be a dynamic process influenced mainly by the integrity of the intestinal barrier. Anyway, the role of Lactobacillus in the gut liver axis is worthy of recognition, and how to grasp its balance point in gut liver axis-related diseases is also worth further exploration.

## The role of Lactobacillus in IBD-related gut-brain axis

With the rise of gut-brain axis research, gut microbiota was emphasized to affect numerous central nervous system activities (CNS) activities. Other than interfering with brain shape in healthy conditions and resting-state brain function, microbiota also labored to commit numerous CNS diseases such as Alzheimer’s Disease (AD), depression, and anxiety [72, 73]. Here, we emphasized the role of Lactobacillus in the IBD-related microbiota-gut-brain axis (MGBA).

### The role of Lactobacillus in memory and cognition

According to several extensive cohort studies, a significant parallel relationship existed between Alzheimer’s disease and IBD. At the same time, the credible explanation may be attributed to NLRP3-dependent neuroinflammation and accumulation of amyloid plaques induced by increased neutrophils [74–76]. As one of the typical symptoms of AD, the decline of memory and cognition has brought great psychological suffering to AD patients and their families. Hopefully, a recent study has uncovered a relationship between the gut microbiome and host memory and cognition in germ-free (GF) mice with supplementary microbiota or not. Specifically, supplementary Lactobacillus reuteri F275, Lactobacillus plantarum BDGP2 and Lactobacillus brevis BDGP6 significantly improved memory capacity compared to uninoculated GF mice, and Lactobacillus plantarum P8, as well as Lactobacillus casei ATCC 393, successfully enhanced cognitive traits of hosts, proclaiming the participation of Lactobacillus in preserving memory & cognition via MGBA [77–79].

To twig the way Lactobacillus acted in MGBA, many genera were tested in experiments for their function mechanism. In healthy mice, the benefit of Lactobacillus acidophilus in ameliorating cognition has already been verified, of which the critical factor was ascertained as butyrate Lactobacillus acidophilus produced [80]. As for the related signaling pathway, in the AD rodent model, ProBiotic-4, a probiotic preparation composed of Bifidobacterium lactis, Bifidobacterium bifidum, Lactobacillus acidophilus, and Lactobacillus casei altered the memory deficits, synaptic and cerebral neuronal injuries, of which the mechanism may be associated with inhibition of NF-κB signaling pathway mediated by TLR4 and RIG-I, with the comprehensive details remained to be explored [81]. What is more critical, CCAAT/enhancer binding protein β/asparagine endopeptidase (C/EBPβ/AEP) signaling pathway, cleaving Tau and β-amyloid precursor protein in the brain and resulting in the pathogenesis of AD, was proved to have existed in the gut of AD mouse model and possess a mutual interaction with AD progression in the brain. However, Lactobacillus salivarius inhibited the above signaling pathway and led to attenuated amyloidogenic processes in the gut, suggesting a probable therapeutic target in AD through MGBA [82].

### The role of Lactobacillus in depression and anxiety

Highly comorbid with IBD and functional bowel disorders, depression and anxiety aroused worldwide concern. Meanwhile, the auxiliary mitigation of Lactobacillus in psychiatric disorders in IBD and other diseases, such as anxiety in the postpartum period, was reported in several clinical studies, with the comprehensive mechanism still requiring elucidation [83–85].

As one of the hypotheses of depression etiology, depression was used to being knitted together with inflammatory cytokines in the hippocampus. In this field, a crucial inflammatory factor, IL-1β, was observed downregulated by Lactobacillus gasseri NK109 with the simultaneous upregulated expression of brain-derived neurotrophic factor (BDNF) in the hippocampus of mice with cognitive impairment and depression induced by Escherichia coli K1, suggesting its alleviation in brain inflammation against neuropsychiatric disorders caused by bacterial infection [86]. Another key player who got involved in managing many physiological and psychological processes, GABA, was a primary inhibitory neurotransmitter in the CNS. In improving the behavioral performance of mice, Lactobacillus rhamnosus JB-1 was included and found to affect the expression of GABA receptors in the brain in a region-dependent manner. To put it more rigorously, Lactobacillus rhamnosus JB-1 was recorded to increase GABA(B1b) mRNA in cortical regions while reducing it in the hippocampus, amygdala, and locus coeruleus. As for GABA(Aα2), the expression was detected reduced in the prefrontal cortex and amygdala while increasing in the hippocampus [87]. As an additional remark, the effect of Lactobacillus rhamnosus JB-1 in depression and anxiety was CD4 + CD25 + T cells dependent [88]. Set aside as neurotransmitters, enzymes got affected, too. As a noteworthy factor whose activity seen to correlate with depression in the cerebral cortex, xanthine oxidase was also inhibited by Lactobacillus paracasei CCFM1229 and Lactobacillus rhamnosus CCFM1228, which also became an essential link between Lactobacillus intervention in depression [89] Expectedly, there also existed researches attributing the protective effect of Lactobacillus to its product such as ergothioneine produced by Lactobacillus reuteri. The oral administration of ergothioneine had a preventative effect on depressive behaviors induced by social defeat stress (SDS) in rodents, especially on sleep abnormalities in the rapid eye movement sleep phase [90]. These alterations indicated the possible mechanism Lactobacillus may hold to affect emotion and behavior.

Moreover, given the benefits humanity could gain from Lactobacillus, researchers began trying to prevent diseases before they happened and made a breakthrough. The early life colonization of Lactobacillus rhamnosus could relieve anxiety-like behavior in adulthood with an accompanying increased abundance of beneficial bacteria such as Akkermansia and Bifidobacteria in rodent intestine, proclaiming the necessity of lactobacillus supplementary in the absence of disease [91].

**Fig. 2:**
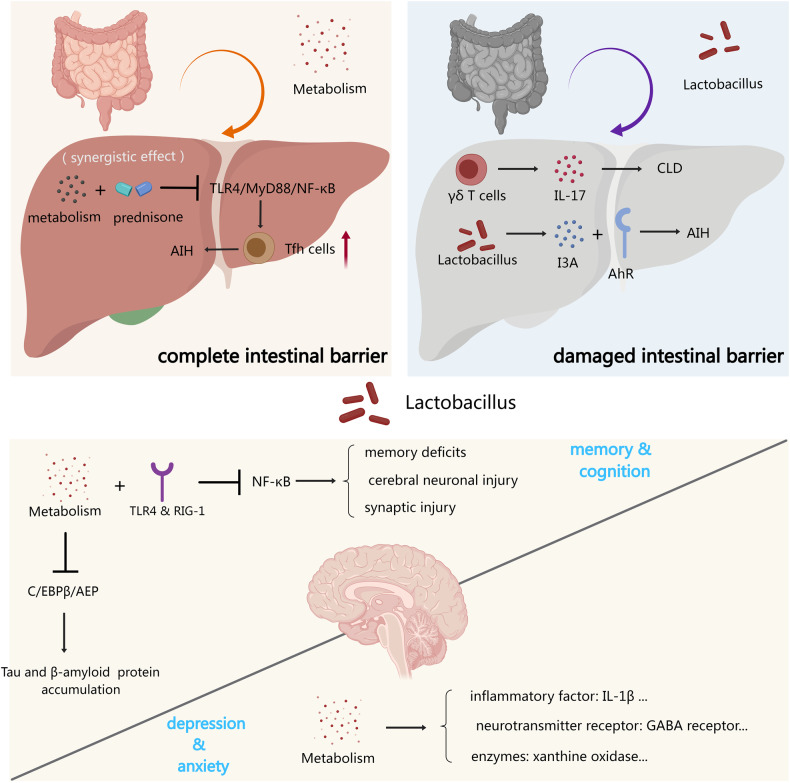
The role of Lactobacillus in the gut–liver axis and gut–brain axis. The role of Lactobacillus in the gut–liver axis was related to the severity of enteritis, which was directly proportional to the integrity of the intestinal barrier. In neurological diseases, due to the presence of the blood–brain barrier, metabolites of Lactobacillus had a certain alleviating effect on cognition, memory, depression and anxiety.

In a word, to portray the role of Lactobacillus in the gut-liver axis and gut-brain axis more intuitively, we exhibited a figure here to describe the process involved (Fig. 2).

## The role of Lactobacillus in CAC

In consideration of the relationship between IBD and Colitis-associated Colorectal cancer (CAC), as well as the anti-inflammatory effect of Lactobacillus, wonders were raised whether Lactobacillus would have the anti-tumor ability in tumorigenesis or therapeutic capability in the treatment of CAC. First but not foremost, an Anti-tumor effect was observed in Lactobacillus bulgaricus, of which the administration inhibited mean tumor size and total tumor volume in azoxymethane (AOM)/ DSS-induced CAC mouse model with remarkably reduced pro-inflammatory cytokines containing IL-6, TNF-α, IL-1β, IL-17, and IL-23 [92]. Similarly, a specific polysaccharide-peptidoglycan complex (PSPG) derived from specific Lactobacillus casei Shirota, but not other strains, was also proclaimed to limit tumor growth via inhibiting the activation of the IL-6/STAT3 signaling pathway [93]. In the aspect of the tumor stage, the anti-proliferation effect of Lactobacillus helveticus NS8 in enterocytes was found to be more efficient at the early stage of CAC, indicating its vital role in the prevention of tumorigenesis [94].

**Fig. 3:**
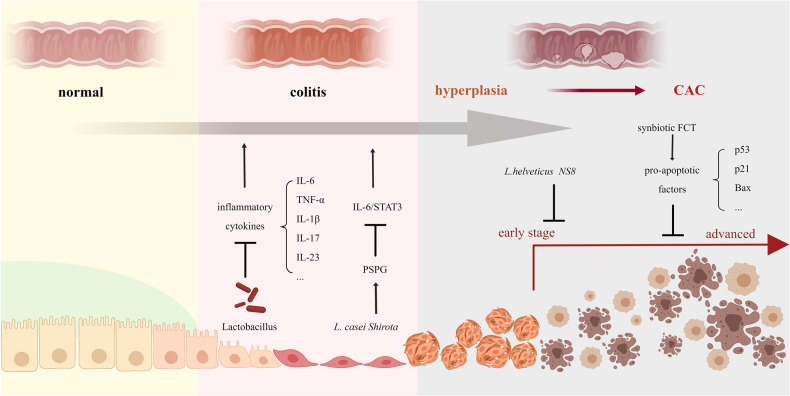
The role of Lactobacillus in CAC. The role of Lactobacillus in CAC varied with disease progression. In the stage of enteritis, it inhibited the expression of inflammatory factors and hindered related signaling pathways. In the stage of dysplasia, it not only hindered the progression of CAC in the early stage, but also promoted tumor cell apoptosis during the advanced stage, thereby delaying the progression of CAC.

Given the anti-tumor effect of single strains, the mixture of lactobacillus and other probiotics was also attached to attention and obtained lots of expected results. A probiotic combination of Lactobacillus acidophilus, Bifidobacterium bifidum, and Lactobacillus rhamnosus showed its potential chemo-preventive effect as it hampered tumor growth in both number and volume in mice with AOM/DSS induced CAC [95]. Compared with probiotics, research on symbiotics has also been a hotspot recently. Synbiotics refers to combining probiotics and prebiotics or adding vitamins, trace elements, etc. Synbiotics can exert the physiological bacterial activity of probiotics and selectively and rapidly increase the number of such bacteria, making the probiotics’ function more significant and lasting [96]. It has been reported that synbiotics offered help in the inhibition of CAC in a research program that claimed that FCT, a synbiotic consisting of Lactobacillus gasseri 505 and cudrania tricuspidata leaf extract, successfully reduced incidence of colonic tumors, as well as damage to the colonic mucosa with significantly downregulated pro-inflammatory cytokines, upregulated TJs and increased pro-apoptotic factors such as p53, p21, and Bax, proclaiming its therapeutic value in CAC [97]. The role of Lactobacillus in CAC is displayed in figure (Fig. 3).

## Lactobacillus-related treatment in IBD and IBD-related diseases

As the same principle as the application of Lactobacillus acidophilus in the fermentation industry, to better take advantage of the anti-inflammatory benefits of Lactobacillus, development in reverse engineering of anti-inflammatory fermented foods specially designed for IBD patients was brought into the agenda. At the laboratory stage, in mice with m2,4,6-trinitrobenzene sulfonic acid (TNBS)-induced colitis, single-strain lactobacillus-fermented milk, as well as an experimental pressed cheese fermented by two strains comprised of Lactobacillus delbrueckii and Propionibacterium freudenreichii, another probiotic featured by producing propionic acid and acetic acid from carbohydrate, was observed to modulate intestinal and systemic inflammation, relieve the severity of symptoms, meanwhile offer adequate protection against not only colonic oxidative stress but epithelial cell damages [98]. In addition, compared with the therapeutic value of a specific single strain, probiotic consortia containing Lactobacillus reuteri, Lactobacillus gasseri, Lactobacillus acidophilus, and Bifidobacterium lactis were demonstrated to better ameliorate DSS-induced colitis in the mouse model, with their mixed metabolites receiving slightly inferior benefits by comparison with the whole strain [99].

As we have put it before, synbiotics, a combination of probiotics and prebiotics, or the addition of vitamins, trace elements, and so on, have been widely used in IBD-related studies recently. Among various synbiotics, vitamin D (VitD) became an emerging component demonstrating rapport with Lactobacillus in relieving intestinal inflammation [100]. To illustrate, VitD levels were usually negatively correlated with IBD activity. However, VitD supplementation alone failed to reduce IBD severity since its effect was limited by the downregulation of vitamin D receptor (VDR), whose downregulation resulted in damaged autophagy in inflammatory status [101]. At this point, Lactobacillus was recorded to restore the hindered expression of VDR in human cell line HCT116 and intestinal organoids, and Lactobacillus rhamnosus GG secreting p40 demonstrate good partnership with VitD in promoting colonic epithelial proliferation and alleviating colitis in mice, proposing a promising combination in clinical application [101, 102].

In the innovative attempt to treat IBD, Fecal Microbiota Transplantation (FMT) has achieved particular success. Surprisingly, the relative abundance of Lactobacillus after antibiotics positively correlated with engraftment in FMT and improved clinical reaction. Therefore, it may be possible to try moderately supplementing Lactobacillus after antibiotic cleaning to enhance the clinical efficacy of FMT and better alleviate colitis in IBD patients.

In recent years, in addition to traditional salicylic acid preparations and hormones, biological agents have greatly alleviated the severity of enteritis in the clinical treatment of IBD patients. Among them, the most commonly used monoclonal antibodies in clinical practice are infliximab, adalimumab, ustekinumab, and vedolizumab. In this area, co-administration of Lactobacillus gasseri and tumor necrosis factor-alpha inhibitor infliximab improved colitis in mice [103]. At a deeper level of clinical application, we have noticed that multiple studies have mentioned the correlation between the efficacy of biological agents and the composition of gut microbiota in patients [104]. However, due to insufficient sample size and limited research cases, there is currently no research to make a judgment on whether Lactobacillus can be used as a biological agent for efficacy prediction, which is precisely what we want to emphasize: more research ought to be invested in evaluating the predictive and monitoring effects of Lactobacillus on IBD.

Considering their remarkable anti-inflammatory benefits, the therapeutic application of Lactobacillus in patients with IBD and IBD-related diseases was on the way. Some of them have already been implemented with a result published and available to the public, and some of them were still in recruiting or even just taken shape. Here, a table that offered an overview of relative clinical trials comprised of projects in progress was listed to facilitate researchers and patients to search for helpful information (Table 2).

**Table 2:** Ongoing clinical trials of Lactobacillus in IBD and IBD-related diseases.

Identifier	Lactobacillus involved	Disease	Age of subject	Enrollment	Location	Study phase
NCT05118919	*L.reuteri BGP-014*	UC	18 years and older	50	Sweden	Recruiting
NCT05652621	*L.reuteri PLBK1*	UC and IBS	18 to 80	200	China	Recruiting
	*L.reuteri PLBK2*					
	*L.reuteri PLBK3*					
	*L.reuteri PLBK4*					
NCT04908644	No clear explanation	UC	20 to 65	40	Taiwan, China	Recruiting
NCT05032014	*L.rhamnosus Probio-M9*	HCC	18 to 70	46	China	Recruiting
NCT05521477	*L.acidophilus DSM 32241*	AD	60 to 80	3	Switzerland	Not yet recruiting
	*L.helveticus DSM 32242*					
	*L.paracasei DSM 32243*					
	*L.plantarum DSM 32244*					
	*L.brevis DSM 27961*					
NCT05266443	*L.acidophillus (LA-5)*	Depression in IBS	18 to 65	140	Malaysia	Recruiting
	*L.paracasei (L. CASEI-01)*					
NCT05717946	*L.helveticus Rosell*	Depression	18 to 70	100	Poland	Recruiting
NCT03660280	No clear explanation	Depression	18 to 85	76	Sweden	Recruiting
NCT04756544	*L.helveticus Rosell*	Depression	18 to 70	200	Poland	Recruiting
NCT05568498	*L. acidophilus W37*	Depression in PD	40 to 80	60	Canada	Not yet recruiting
	*L. brevis W63*					
	*L. casei W56*					
	*L. salivarius W24*					
NCT04772664	*L.acidophilus W22*	Depression	18 to 65	80	Austria	Recruiting
	*L.casei W56*					
	*L.paracasei W20*					
	*L.plantarum W62*					
	*L. salivarius W24*					

## Conclusions and prospects

Undoubtedly, as the most common probiotics, Lactobacillus played a vital role in maintaining the ecological balance of intestinal flora since it exerted excellent anti-inflammatory effects in IBD and IBD-related intestinal diseases such as CAC, as well as CNS diseases and liver diseases represented by AD, PD, and AIH, in both mouse model and clinical patients ranging from children to adults. As a symbiotic existence with the host, it helped to maintain the immune microenvironment in the intestinal mucosa, confine the over-activation of inflammatory signals, and safeguard IECs from multifarious threats to support patients in overcoming the intestinal inflammatory storm. Though sharing numerous resemblances, each stain’s anti-inflammatory principle and functional component were not identical because of the specific characteristics of different subspecies. Therefore, the combined utilization of different strains of Lactobacillus to develop more efficient probiotics or to jointly use Lactobacillus with other probiotics or prebiotics will become a powerful adjuvant therapy to meet the requirement of patients suffering from IBD and IBD-related diseases. In this regard, emerging microorganisms in recent years, such as Akkermansia muciniphila, Ruminococcus gnavus, and so on, may be called powerful candidates to deliver a partnership with Lactobacillus, which still demands experimental verification.

With the development and popularization of various new technologies, multiple sequencing techniques have gradually been used to study gut microbiota, including Lactobacillus. To cite a case, a combination of global RNA sequencing of human biopsies and bacterial DNA sequencing was carried out to precisely describe the effects of Lactobacillus rhamnosus GG on the human intestine, displaying an activation and proliferation of existing B cells in jejunum and proposing that the treatment of probiotics should be individualized [105]. In addition, to better describe the effect of exogenous prebiotics on overall gut microbiome stability in probiotic consumption, shotgun metagenomic sequencing successfully pointed out that continuous galactooligosaccharide supplement could promote the growth of Lactobacillus plantarum and decreased its single-nucleotide polymorphisms (SNPs) mutation under competitive conditions [106]. In brief, sequencing techniques have become a method that can accurately reflect the relationship between probiotics and the host gut immune microenvironment.

In the author’s view, although Lactobacillus has been proven to have many anti-inflammatory effects in mice, its clinical efficacy is still hindered by issues such as low vitality and low bioavailability in gastrointestinal transportation. Research in Ligilactobacillus salivarius developed a new packaging method for probiotics, which means layer-by-layer (LbL) encapsulating a single bacterium with chitosan and alginate to tremendously increase the potential of Ligilactobacillus salivarius in alleviating colitis [107]. Moreover, an adhesive core-shell hydrogel microsphere, colon-targeted, was fabricated by advanced gas-shearing technology and ionic diffusion method to prolong the local drug dwell time. Thus, we eagerly anticipate applying similar technologies in the colon-targeted transportation of Lactobacillus to improve their bioavailability [108]. Therefore, while we focus on the rational combination of probiotics with probiotics or prebiotics, an effective packaging method for probiotics seems to double our efforts with half the effort.

In the end, we emphatically proclaim the magnitude of Lactobacillus in the gut-liver axis and gut-brain axis and appeal for more attention on probiotics in communication between multiple systems, which will provide more options for relieving patients with IBD-related diseases from affliction.
